# An unusual case of osteoarthritis?

**DOI:** 10.1002/ccr3.4662

**Published:** 2021-08-21

**Authors:** Mariana Teixeira, Joana Ricardo Pires, Filipa Coroado Ferreira, Tiago Rabadão, Marcelo Aveiro, Nuno Neves, Clarinda Neves

**Affiliations:** ^1^ Internal Medicine Department of Centro Hospitalar do Baixo Vouga Aveiro Portugal; ^2^ Imaging Department of Centro Hospitalar do Baixo Vouga Aveiro Portugal

**Keywords:** bone, diffuse idiopathic skeletal hyperostosis, Forestier disease, osteoarthritis, radiography

## Abstract

Forestier disease is a condition characterized by calcification and ossification of ligaments and entheses particularly affecting axial skeleton. Diagnosis is difficult and mandates a high suspicion level, but unexpensive and accessible examinations like a simple radiography might provide useful diagnostic clues in these challenging clinical scenarios and improve clinical assistance.

## Graphical abstract

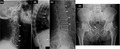

## 1 CLINICAL IMAGE

A 70‐year‐old man with history of hypertension presented with difficulty in mobilization of his head and neck during the previous months, with hypoesthesia on the left portion of the forehead, limitation while walking due to loss of strength in the dorsiflexion, hypoesthesia, and paresthesia of the left foot. He presented with a sedimentation rate of 85 mm (<15 mm), rheumatoid factor, anti‐CCP, and HLA‐B27 were negative. The images of the skeletal radiography (Panels A‐D) showed an ossification of the ligaments and muscle insertions, particularly alongside the cervical and lumbar portion of the anterior longitudinal ligament (Panels A‐C) and diffuse signs of enthesopathy, the most notorious at the pubic symphysis (Panel D). These findings suggested the diagnosis of diffuse idiopathic skeletal hyperostosis (DISH). Pain management was initiated.

The prevalence of DISH is expected to rise as it is related to older age and metabolic syndrome.^2^ The pelvis is one of the main extraspinal involved sites and has been suggested to be included in future classification criteria.^1^ The clinical relevance is beyond unstable spinal fractures, to the involvement of cardiovascular, respiratory, and gastrointestinal systems^1^, ^2^—a computed tomography angiography of the cardiac, cervical, and cerebral vasculature was ordered during the follow‐up, to assess systemic involvement.

## CONFLICT OF INTEREST

The authors declare that they have no conflict of interest.

## AUTHOR CONTRIBUTIONS

MT conceptualized the study, acquired and analyzed the data, and drafted the manuscript. JRP, FCF, TR, and MA conceptualized the study, drafted the manuscript, and reviewed the manuscript. CN and NN supervised the drafting of the manuscript and reviewed the manuscript. All authors reviewed the final draft of the manuscript and approved its submission.

## ETHICAL APPROVAL

Compliance with ethical standards.

2

**FIGURE 1:**
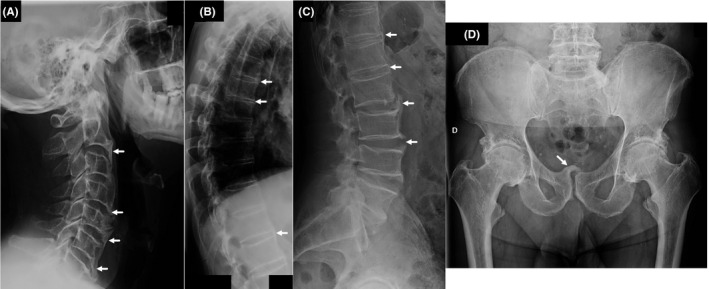
A: Cervical spine radiography revealing thickening and ossification of anterior longitudinal ligament (arrows). B: Thoracic spine radiography revealing ossification of anterior longitudinal ligament (arrows). C: Lumbar spine radiography revealing large nonmarginal osteophytes (arrows). D: Pubic symphysis enthesopathy (arrow)

## Data Availability

Data sharing is not applicable to this article as no new data were created or analyzed in this study.
